# Structure and Function of Enterocyte in Intrauterine Growth Retarded Pig Neonates

**DOI:** 10.1155/2017/5238134

**Published:** 2017-07-05

**Authors:** Karolina Ferenc, Tomasz Pilżys, Tomasz Skrzypek, Damian Garbicz, Michał Marcinkowski, Małgorzata Dylewska, Paweł Gładysz, Oleksandr Skorobogatov, Zdzisław Gajewski, Elżbieta Grzesiuk, Romuald Zabielski

**Affiliations:** ^1^Department of Physiological Sciences, Warsaw University of Life Sciences, Nowoursynowska 100, 02-797 Warsaw, Poland; ^2^Veterinary Research Centre, Department of Large Animal Diseases with Clinic, Faculty of Veterinary Medicine, Warsaw University of Life Sciences, Nowoursynowska 100, 02-797 Warsaw, Poland; ^3^Department of Molecular Biology, Institute of Biochemistry and Biophysics, Polish Academy of Sciences, Pawińskiego 5a, 02-106 Warsaw, Poland; ^4^Interdisciplinary Research Center, The John Paul II Catholic University of Lublin, Lublin, Poland

## Abstract

The intestine of intrauterine growth retarded (IUGR) neonates showed different morphology compared to neonates born with normal body weight (NBW). The aim of the present study was to investigate the ultrastructure and proteomic profile of the gut epithelium in IUGR pig neonates with special attention to the digestive and absorptive function. Intestine tissue samples were investigated in 7-day-old IUGR and NBW littermate piglets using histometry, immunofluorescence, scanning electron microscopy (SEM), and mass spectrometry analysis. IUGR piglets have shown reduced mucosa and muscularis thickness and an enhanced number of foetal type enterocytes (FTE). SEM studies have shown the lack of the characteristic large-size vacuole in IUGR's enterocytes. Delayed removal of FTE in IUGR neonates was probably due to the inhibited apoptosis in the apical part of villi and increased apoptosis and reduced mitosis in the crypt region. In the expression of proteins in the intestinal mucosa such as hexokinase I, histones, and prelamin A/C, carbamoyl phosphate was reduced in IUGR neonates. Finally, IUGR intestines showed higher expression of HSPA9 and HSPA5 as apoptosis markers. The data indicate modifications of gut mucosa in IUGRs that may result in slower gut mucosa maturation and reduced utilisation of nutrient as compared to NBW pig neonates.

## 1. Introduction

Intrauterine growth retarded (IUGR) newborn piglet is born on time, and it is characterised by low birth body mass (below 1.1 kg), high perinatal mortality, and a “dolphin-like” head shape compared with “normal” piglets [1, 2]. The abnormality of head morphology is explained by prioritized brain development due to the “brain sparing effect” as part of a foetal adaptive reaction to placental insufficiency [3]. In IUGRs, also a catch-up-growth (CAG) is observed, which is the time when neonates are able to compensate their low body birth weight by accumulation of adipose tissue in visceral area, rather than by muscle mass growth.

Since nutrients in IUGR foetuses are being allocated preferentially to the brain, the development of other organs is compromised. The digestive system in IUGR neonates showed a number of alterations, both on a tissue and molecular level. In brief, modifications in gut development included smaller size and weight [4], delayed maturation of intestinal mucosa [5] as well as impaired intestinal motility, and digestion and absorption of colostrum and milk [5, 6]. Brush border enzyme activity was markedly affected, and the transfer of macromolecules from the gut to blood circulation was enhanced [5, 6]. Proteomic studies by Wang et al. [7] revealed that cellular signalling defects, redox imbalance, reduced protein synthesis, and enhanced proteolysis could be major mechanisms responsible for abnormal absorption and metabolism of nutrients, as well as reduced growth and impaired development of the small intestine, liver, and muscle in IUGR neonates. The more recent paper by Wang et al. [8] indicates that small intestinal mucosal permeability and mRNA expression of redox-sensitive genes is affected in IUGR piglets.

The supply of energy, via glycogen mobilization and colostrum, is of major importance for the neonate piglet [9, 10] until abundant milk production begins ~33 h after onset of parturition [11]. At least 200 g of colostrum per piglet is required to maintain life during the neonatal phase [9]. Amdi et al. [12] demonstrated that only “normal” piglets ingested the right amount, as opposed to IUGR piglets. This was further supported by decreased plasma glucose levels and lower remaining glycogen depots in the liver in IUGRs at 24 h. Sugar transport in IUGR enterocytes was not studied in detail. Nonetheless, some IUGR piglets died with a full stomach suggesting dysfunction at the level of the small intestine digestion and/or absorption. Previous histology studies showed that foetal-type enterocytes (FTE) remain much longer in IUGR piglets, in particular in the lower half of the small intestine as compared to “normal” pig neonates. Moreover, a clear-cut organization of FTE, namely, numerous vesicles and cisterns in the apical region and one large-size vacuole in the centre, was lost [5]. The foetal-type enterocytes are responsible for both macromolecule absorption and intracellular digestion, since in neonates, the secretion of digestive juices is of low potential [13]. The aim of this study was to investigate the enterocyte structure and function in IUGR neonatal piglets with special attention to digestive and absorptive functions that may help to understand the mentioned differences between IUGR and normal neonates.

## 2. Materials and Methods

### 2.1. Animals, Tissue Collection, and Histological Analyses

The protocol was conducted in compliance with the European Union regulations concerning the protection of experimental animals. The study protocol was approved by the Local Ethical Committee, Warsaw University of Life Sciences, Warsaw, Poland. Briefly, 8 pairs of neonatal piglets (*Sus scrofa domesticus*, Landrace × Pietrain) of both sexes, each pair selected from a different litter, were used in the study. Pairs were selected as follows: one piglet was of normal birth body weight, that is, representing the average weight of all littermates (between 1.3 and 1.6 kg) from livestock, and the other one was of low birth weight between 0.6 and 0.9 kg, recognized as asymmetric IUGR with the characteristic head [13] and spontaneous background.

Sows were kept on a standard diet during pregnancy (dry matter (DM) 87.6%, mean energy (ME) 11.35 MJ/kg, and crude protein (CP) 13.1%) and lactation (DM 87.3%, ME 12.93 MJ/kg, and CP 15.4%). Fresh diet and water were provided daily ad libitum. Piglets were delivered at term and were clinically healthy. On the postnatal day 3 (PD3), all piglets were injected intramuscularly with 100 mg iron dextran (FeDex, Ferran100, 10% solution, Vet-Agro, Lublin, Poland). NBW and IUGR piglets were kept together with their litters and fed by the sow until postnatal day 7 (PD7). On that day, the NBW and IUGR piglets were taken from sow, killed by barbiturate overdose, and exsanguinated, and the entire gastrointestinal system and brain were gently removed and measured for weight and size.

Small intestine (SI) tissue samples from the duodenal, proximal, middle, and distal part of the jejunum and ileum whole-thickness segments (3 cm) were fixed in 4% buffered formaldehyde and then stored in ethanol. Furthermore, from a 15 cm segment of the middle part of the jejunum, the mucosa was scraped and the homogenates of intestinal epithelium were frozen in −80°C for future analysis.

### 2.2. Histological Studies

The samples were embedded in paraffin, sliced in 5 *μ*m sections, and mounted on a microscopy glass. Deparaffinisation of slides included two washes in xylene for 15 min and rehydration in decreasing concentrations of ethanol (from 100% to 70%). Serial histological 5 *μ*m sections were stained with hematoxylin and eosin for morphometric analysis under the light microscope. Morphometric analysis included measurements of the length of villi, crypt depth, mucosal thickness, and muscle layer thickness, as well as the presence of large vacuoles, as markers of enterocyte maturation. Five to ten slides for each tissue sample were prepared, and 30 measurements were performed using an optical binocular microscope (Olympus BX60; Olympus, Warszawa, Poland) coupled via a digital camera to a personal computer equipped with a Cell^P (Olympus) software.

### 2.3. Scanning Electron Microscopy (SEM)

Formaldehyde-fixed sections were washed in saline and dehydrated in a series of alcohol solutions. After drying in a critical point drier, samples were sputter coated with layer of gold-palladium (Au/Pd) and examined using Ultra Plus (Zeiss) SEM for complete description of the method refer to Skrzypek et al. [14].

### 2.4. Immunofluorescence Studies

After paraffin fixing, tissue samples were sliced into 5 *μ*m sections and rehydrated. Antigen retrieval was performed by boiling the slides in citrate buffer. Nonspecific binding was blocked with 1% BSA (Sigma) in PBS at room temperature for1 h. Samples were labelled with specific antibodies against active caspase-3 (FITC-conjugated, BD Pharmingen) or lamin A/C (unconjugate, Santa Cruz Biotechnology INC, sc-20681) at 1 : 100 ratio with 0.1% BSA in PBS by overnight incubation at 4°C. For antilamin A/C antibody, 2 h incubation with secondary antibodies (Thermo Fisher Scientific AlexaFluor 568, A-11011) in concentration 1 : 200 was performed at room temperature. Cell nuclei were stained with Hoechst 33342 at 10 *μ*g/ml concentration for 30 sec at room temperature (Life Technologies). Slides were mounted in Fluoromount Aqueous Mounting Medium (Sigma). Sequence scanning was used to omit cross-talk between fluorescent dyes. Confocal microscopy (Olympus FV500, objective 20x) was employed for in-tissue cytometry analysis. Ten images per one tissue sample were made. Quantification of apoptotic cells was performed according to in-tissue cytometry procedure [15] by measurement of active caspase-3 expression.

### 2.5. Proteomics Analysis

Prior to protein extraction, frozen samples were homogenized in liquid nitrogen. The resulting powder was extracted with RIPA buffer (Sigma-Aldrich, RO278) in the presence of a protease inhibitor cocktail (Sigma-Aldrich, P8340) supplemented with EDTA and PMSF to inhibit protein degradation. During extraction, tissues were further homogenized. Cellular debris was spun down at 30,130 g. Samples were diluted with SDS-PAGE loading buffer, and pellets were suspended in SDS-PAGE loading buffer. Samples were loaded on the Mini-PROTEAN TGX 4–15% gradient gels (Bio-Rad). Then, total protein was standardized in 3 steps: (i) equal masses of the tissue were taken for extraction in RIPA buffer (50 mg); (ii) the extract was then assayed by Bradford for protein content; and (iii) equal amounts were loaded on the gel and verified by Coomassie staining. Samples were analysed by liquid chromatography coupled to tandem mass spectrometry (LC-MS-MS/MS) using the nanoACQUITY (Waters) LC system and Orbitrap Velos mass spectrometer (Thermo Electron Corp). Proteins were subjected to standard “in-gel digestion” with proteins reduced with 50 mM TCEP (for 60 min at 60°C), alkylated with 200 mM MMTS (for 45 min atRT), and digested overnight with trypsin (sequencing Grade Modified Trypsin, Promega). Peptide mixtures were applied to RP-18 precolumn (nanoACQUITY Symmetry C18, Waters) using 0.1% TFA as a mobile phase and then transferred to nano-HPLC RP-18 column (nanoACQUITYBEHC18, Waters) using the acetonitrile gradient (5–35% AcN in 180 min) in the presence of 0.05% formic acid with the flow rate of 250 nl/min. Column outlet was directly coupled to the ion source of the spectrometer working in the regime of data-dependent MS to MS/MS switch. A blank run ensuring lack of cross contamination from previous samples was included.

Acquired raw data were processed by Mascot Distiller followed by Mascot Search (Matrix Science) against UniProt (v. 201604) database restricted to *Sus scrofa* sequences. The following search parameters were used: precursor and product ion mass tolerance of 20 ppm and 0.1 Da, respectively; trypsin as the enzyme specificity; 1 missed cleavage site allowed; fixed modification of cysteine by methylthiol; and variable modification of methionine oxidation. Peptides with Mascot Score exceeding the threshold value corresponding to <5% expectation value and FDR < 1%, calculated by Mascot procedure, were considered as positively identified.

### 2.6. Western Blot Analysis

Western blot analysis was performed with specific primary antibodies used at dilution 1 : 1000 polyclonal anti-HXKI (Santa Cruz Biotechnology INC, sc-6517), polyclonal anti-GRP78 (Santa Cruz Biotechnology INC, sc-13968), and polyclonal antilamin A/C (Santa Cruz Biotechnology INC, sc-20681). Appropriate secondary antibodies anti-goat (Thermo Scientific, 31402) and anti-rabbit (Santa Cruz Biotechnology INC, sc-2054) conjugated with horse-radish peroxidase usedat dilution of 1 : 5000. All incubations were performed in 5% milk/PBST. Chemoluminescence was measured using the ChemiDoc MP Imaging System (BioRad).

## 3. Statistical Analysis

The results were subjected to two-stage statistical analysis. In the first step, we checked the uniformity of the SD and Kolmogorov-Smirnov *A* test of the normal distribution. Depending on the outcome, the test data were then analysed with an unpaired *t*-test or test of the *t*-test and Welch (normal distribution) or Mann–Whitney *A* test (in its absence). All statistical analyses were done using the GraphPad Prism v.5.0 (GraphPad Software, CA, USA); *p* < 0.05 was considered significant, *p* < 0.01 highly significant, and *p* < 0.1 a trend.

## 4. Results

The asymmetric IUGR syndrome was confirmed in pig neonates by low birth body weight, head shape, brain weight (Table 1), and clinical examination at birth. The IUGR piglets increased the body weight by ca. 10% during the first week of life as the NBW piglets did. At PD7, the body weight, liver weight, and small intestine weight and size were significantly lower in IUGR piglets as compared to their NBW littermates (Table 1).

Histological analysis of IUGR versus NBW showed decreased height of villi in the duodenum and proximal part of the intestine and decreased thickness of muscularis of the jejunum (Table 2).

**Figure 1:**
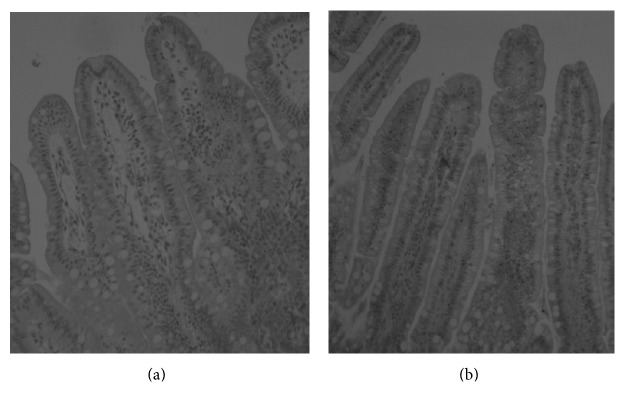
Differences between structure of FTE in NBW (a) and IUGR (b) piglets in the ileum. Lack of characteristic one large vacuole in IUGRs.

The abundance of foetal-type enterocytes, containing large-size vacuoles, in 7-day-old NBW piglets followed the pattern observed repeatedly [14, 16]. Namely, no vacuolated enterocytes were observed in the upper small intestine, whilst in the distal small intestine and ileum, respectively, 71% and 95% of enterocytes contained large-size vacuoles. In contrast, in IUGR neonates, there were still 3 to 4% of vacuolated enterocytes in the duodenum and proximal jejunum, respectively, and 10% in the midjejunum. In the distal jejunum and in the ileum, 84% and 74% enterocytes contained vacuoles, respectively. In the previous studies, in 7-day-old suckling NBW piglets, it was possible to measure the area of each large-size vacuole of FTE using planimetry software, whereas in the present IUGR neonates, it was not. The contour of the vacuoles was unclear (Figure 1).

**Figure 2:**
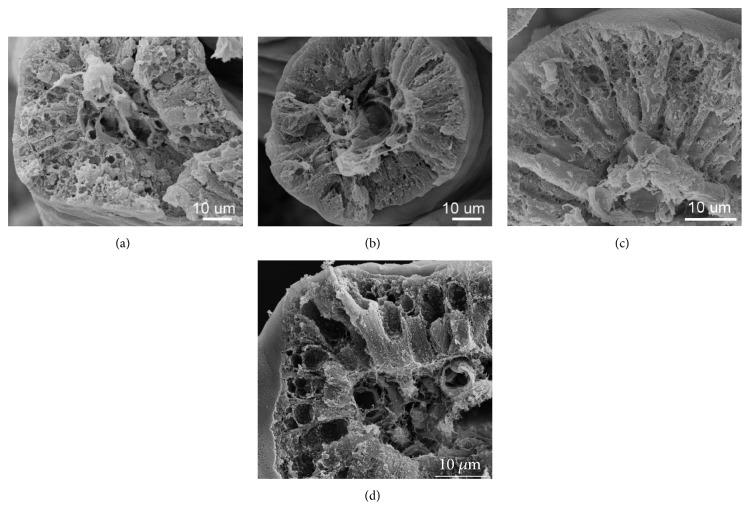
Representative SEM micrographs of the villi in the distal jejunum of IUGR (a, c) and NBW (b, d) piglets on postnatal day 7. The enterocytes of IUGR piglets show numerous small, bubble-like empty spaces (traces of small-size vacuoles) in the upper part of the enterocyte body; however, no traces of large-size vacuoles typical for foetal-type enterocytes are observed (a, c). The micrographs from NBW piglets (b, d) show villi covered by mature enterocytes, no empty spaces of neither large-size nor small-size vacuoles. Horizontal bars depict the scale.

SEM examinations unravelled peculiar ultrastructure of enterocytes lacking characteristic large-size vacuoles in the central part of the enterocyte in IUGRs as compared to NBW piglets. In IUGR piglets, numerous small vacuoles composing a foamy structure instead of one large-size vacuole were observed (Figure 2). It is noteworthy to emphasize that both light microscopy and SEM images showed that the epithelial cell integrity in IUGR piglets is maintained in all small intestinal segments analysed like in non-IUGR littermates.

**Figure 3:**
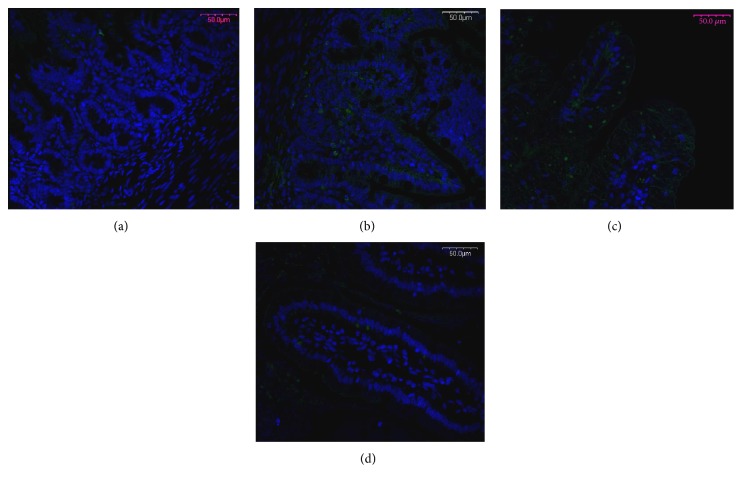
Apoptosis evaluated by the abundance of active caspase-3 expression, in the distal part of the jejunum in NBW (a, c) and IUGR (b, d) and 7-day-old piglets in the crypts (a, b) and in the apical part of the villi (c, d). Apoptosis in IUGRs is decreased in the apical part of the villi and increased in the crypt area as compared to NBW. Green fluorescence (Alexa Fluor 488)—caspase-3 expression; blue fluorescence (Hoechst 33342)—cell nuclei. Objective 20x.

Examination of apoptosis evaluated by caspase-3 expression in the distal jejunum has shown the differences between IUGR and NBW littermates both on the top of the villi and in the crypts. Apoptosis intensity in the apical part of villi was significantly decreased (IUGR = 10.5 ± 2.5 versus NBW = 54.2 ± 6.5; *p* < 0.001), whilst in crypts, the opposite effect was observed (IUGR = 10.2 ± 0.9 versus NBW = 4.5 ± 0.7; *p* < 0.001) (Figure 3).

**Figure 4:**
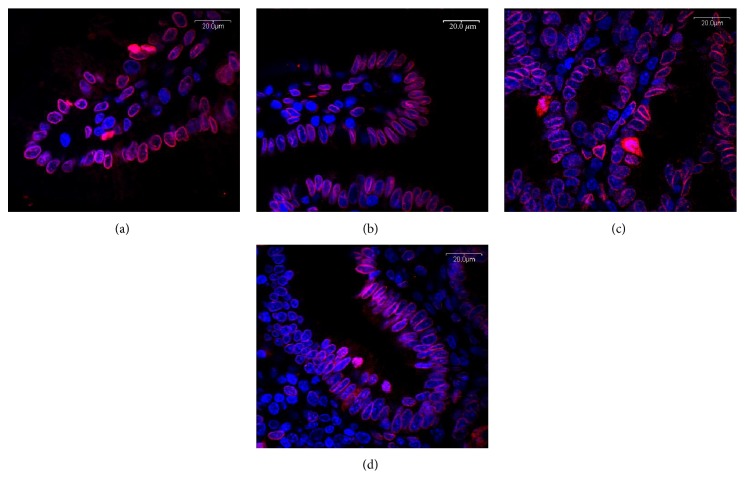
Lamin A/C expression in the middle part of the jejunum in NBW (a, c) and IUGR (b, d) and 7-day-old piglets in the apical part of the villi (a, b) and in crypts (c, d). Apoptosis in IUGRs is decreased in the apical part of the villi and increased in the crypt area as compared to NBW. Red fluorescence (Alexa Fluor 568)—lamin A/C expression; blue fluorescence (Hoechst 33342)—cell nuclei. Objective 100x.

Expression of lamin A/C in IUGRs visualized by confocal microscopy has been decreased both in the apical part of the villi and in crypts (Figure 4).

**Figure 5:**
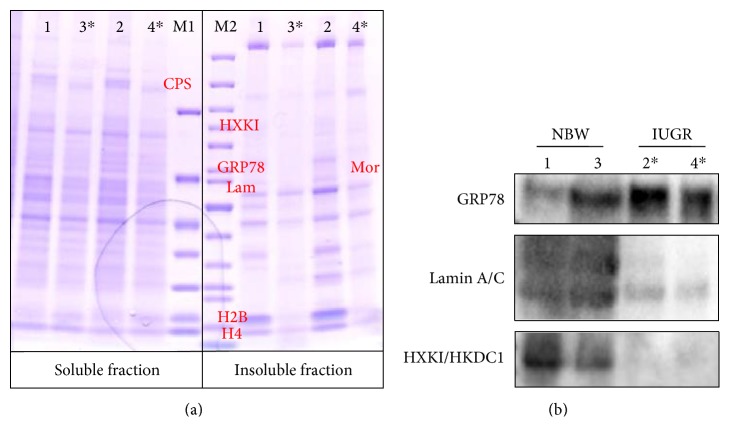
(a) Proteome profile of IUGR in middle part of the jejunum after electrophoresis visualized by Coomassie staining (M1PageRuler unstained protein ladder number 26614, 10–200 kDa, M2—Pierce™ unstained protein MW marker number 26610, 14.4–116 kDa). Proteins identified by mass spectrometry were marked: CPS—carbamoyl phosphate 1, HXKI—hexokinase I, GRP78—78 kDa glucose-regulated protein precursor, Lam—lamin A/C, Mor—mortalin, H2B—histone 2B, H4—histone. (b) Confirmation of mass spectrometry identification by Western blot analysis. IUGR (2, 4 indicated with ∗) in comparison to NBW piglets (1, 3).

Proteomic profile visualized by Coomassie staining showed changes in the expression of several proteins. Main changes were confirmed by Western blot analysis (Figure 5).

Mass spectrometry studies of the mucosa within the middle part of the intestine showed decreased level of proteins involved in gene uptake, chromatin organization, and carbohydrate metabolism in IUGRs compared to NBW littermates. However, we found increased levels of proteins involved in apoptosis and protein metabolism (Table 3).

## 5. Discussion

Individuals displaying IUGR syndrome, due to modification of their energy metabolism in the foetal period and onward, develop metabolic diseases (such as obesity, hyperlipidemia, insulin resistance, and cardiovascular diseases) with much higher probability than the non-IUGR individuals [17]. The problem is serious, also in humans, since it concerns 6–8% of human population [18]. Animal models, including laboratory rodents and pigs, are used to investigate the postnatal development of individuals with IUGR syndrome. Early fat tissue development might be the reason for predisposition of IUGRs to develop metabolic syndrome in childhood and later life [18, 19]. Therefore, IUGR piglets may be also a valuable model for studying early stage of predisposition to development, connected with FTO protein obesity, or type 2 diabetes development [20, 21].

Our morphology studies have shown that IUGRs' absorptive and digestive capabilities can be markedly limited due to the significant decrease in the length of the small intestine, and shortening of the villi within the proximal part of the intestine, where digestion mainly occurs. Other authors have shown reduced height of villi also in the distal small intestine in neonatal piglets [5, 22]. It is important to point out that in our study, we observed similar tendencies also in the distal small intestine; however, they did not reach the statistical significance. One reason for such discrepancies can be the fact that IUGR syndrome in our piglets was developed spontaneously. In our study, pregnant sows were fed accordingly to the requirements and remained clinically healthy during the pregnancy, whereas in the other two studies, IUGR syndrome was induced by extreme pregnant sow diet compositions in regard to energy and protein. Moreover, significant differences in the muscularis of the small intestine were shown (Table 2). Thinner muscularis, notably in the IUGR jejunum, compared to NBW piglets helps to explain altered motility of IUGR intestine [23].

The disappearance of FTE is strictly associated with the maturational processes in the gut mucosa. The timing of disappearance is a good method to precisely define the stage of intestinal mucosa development. Ultrastructure studies of small intestinal epithelium have also shown differences in IUGR neonates' enterocyte structure especially in the distal jejunum and in the ileum. It was already demonstrated that IUGR small intestine maturation has been delayed in comparison to that in non-IUGR littermates. Namely, Mickiewicz et al. [5] have shown the presence of FTE in IUGRs as late as on postnatal day 28. In contrast, in NBW piglets such FTE disappear before postnatal day 14 in midjejunum and before postnatal day 21 in the ileum [14]. The main negative outcome of delayed intestinal mucosa maturation is the increase in difficulty adapting to food changes and enhanced permeability of the intestine for acquisition of alimentary pathogens [8, 22, 23]. This fact may be one of the main reasons of high mortality in IUGRs due to a diarrhea occurrence [24].

Previous electron studies on unsuckling newborn IUGR piglets have indicated the presence of damaged, shorter microvilli and the presence of autophagosomes and swelled mitochondria as compared to NBW piglets [25]. We could not confirm the presence of damaged microvilli in the previous [5] and present studies. The discrepancies are presumably due to the different methods of handling tissue samples and histological processing. However, our SEM studies have demonstrated evident changes in the architecture of an FTE in IUGR neonatal piglets. No apical canalicular system (ACS) structure as well as no single large-size vacuole was observed (Figure 2). Instead, numerous small-size vacuoles were found in the upper and central parts of the cell in each foetal-type enterocyte. To our knowledge, this is the first report showing morphological differences between IUGR and NBW foetal-type enterocytes in neonatal pigs. This striking finding needs further ultrastructure and immunohistochemistry studies, since we expect varied expression of certain cell proteins responsible for ACS forming and fusing smaller vacuoles to one large-size vacuole. Since the large-size vacuoles in the foetal-type enterocytes are important for intracellular digestion of nutrients, we hypothesize that this may be a factor of altered digestion in IUGR neonates. Large-size vacuoles in FTE merge with lysosomes and continue the digestion of nutrients taken up from the gut lumen. We speculate that lysosomal enzymes in IUGRs may not reach all of the small-size vacuoles, thereby intracellular digestion is reduced. Results obtained by Amdi et al. [12] indicating low plasma glucose levels in IUGR neonates strongly support our hypothesis.

In general, apoptosis in IUGR neonates' small intestinal mucosa is increased [5, 22, 26]; that is why high mortalin expression in homogenized intestinal tissues crappings in IUGRs was expected. However, our results on separate intestinal regions, for the first time, have indicated that measured by caspase-3 expression of the apoptosis intensity in the apical part of villi was significantly lower in contrary to the crypt region, where it was elevated. Low apoptosis on the top of the villi suggests delay in the removal of old enterocytes and indicates that the maturation process is diminished. The rationale for apoptotic processes in the crypt region is to eliminate mistakenly divided and/or differentiated, or surplus, cells. We speculate that it may be one of the key factors of delayed maturation of intestinal epithelium being a feature of IUGR individuals. Increased apoptosis in crypt areas may also influence the integrity of intestinal mucosa [27] and, as a result, increased permeability of gut barrier, though in our SEM studies, the integrity of the epithelium was maintained, besides the areas of apoptosis where a number of “unzipped” spaces between dying and living cells can be seen [14].

Our SDS-PAGE electrophoretic separation and mass spectrometry (MS) studies for the first time showed lower levels of expression for HXKI and HKDC1 in IUGR as compared to NBW neonates. Low expression of hexokinases being the first enzyme in the glycolysis pathway is most likely important even for the whole metabolism of enterocytes. Moreover, it has been shown that changes can affect the organization and packing of the chromatin, that is, histones H2B and H4, leading to altered gene expression. It was demonstrated that the histone modification pattern may be altered by the overall availability of amino acids and micronutrients during pregnancy. The maternal nutrition profile may not only influence the foetal programming of postnatal disease susceptibility but genomic imprinting as well [28–30].

Reduced mitosis in the intestinal crypts of IUGR piglets observed previously [5] could be explained by the decreased intensity of lamin A/C. Expression of lamin A/C is a precursor of the process involved in chromatin organization during mitosis. We believe that the increased apoptosis rate within crypt regions can be a consequence of mortalin upregulation which, in a complex with p53 protein, participates in directing cells into programmed death (apoptosis) pathways. For alterations in lamin A/C, histone 2B expression levels have also been described by Wang and coauthors [30].Wang et al. [30] have also found changes in carbamoyl phosphate 1 expression in IUGRs. Carbamoyl phosphate is produced by CPS from one molecule of bicarbonate, two molecules of Mg^2+^ATP, and one molecule either glutamine or ammonia [31]. Decreased expression of CPS in IUGR mucosa influences house-keeping processes including biosynthesis of basic cellular compounds like arginine or pyrimidine nucleotides [32]. CPS is critical in the detoxification of ammonia excess.

## 6. Conclusion

We have managed to detect structural and molecular alterations within the IUGR's intestine, which can lead to pathological aberrations in a number of vital processes such as creation of the intestinal barrier, absorption, and digestion (notably sugars) which may take a part in the catch-up process. On the other hand, these changes might not only affect the homeostasis in IUGR neonates but also have an impact on metabolism in adults.

## Figures and Tables

**Table 1:** Features differentiating NBW and IUGR neonatal piglets. Mean and SD, *n* = 7.

	NBW	IUGR	*p* value
Body weight, PD0 [kg]	1.52 ± 0.17	0.75 ± 0.08	0.002^1^
Body weight, PD7 [kg]	1.66 ± 0.48	0.82 ± 0.19	0.0006^1^
Brain weight, PD7 [g]	30.1 ± 3.06	27.2 ± 4.56	0.3^2^
Liver weight, PD7 [g]	85.6 ± 14.7	38.0 ± 11.2	0.002^2^
Intestine weight, PD7 [g]	83.9 ± 21.8	46.0 ± 10	0.005^2^
Intestine length, PD7 [cm]	369 ± 44	292 ± 26	0.002^2^
Intestine weight/brain weight, PD7	2.72 ± 0.75	1.64 ± 0.56	0.015^2^

PD0, PD7—postnatal days 0 and 7; ^1^nonparametric Mann–Whitney test; ^2^Student's *t*-test.

**Table 2:** Histological analysis (mean ± standard deviation; *n* = 7, nm) of 7-day-old piglet intestine, NBW, and IUGR. Statistical studies were made by two-way ANOVA analysis of variances with Gaussian distributions. This assumption is tested using the method of Kolmogorov and Smirnov.

	Villi			Crypts			Mucosa			Muscularis		
	NBW	IUGR	*p*	NBW	IUGR	*p*	NBW	IUGR	*p*	NBW	IUGR	*p*
DUO	661 ± 162	442 ± 91	0.001	179 ± 50	134 ± 24	<0.1	1191 ± 253	834 ± 216	NS	218 ± 122	179 ± 56	NS
PROX	886 ± 346	552 ± 76	0.001	133 ± 28	106 ± 26	<0.1	1086 ± 211	802 ± 124	NS	183 ± 27	135 ± 11	0.001
MID	833 ± 215	570 ± 108	0.1	113 ± 13	111 ± 18	NS	1319 ± 232	732 ± 84	0.1	190 ± 27	136 ± 22	0.001
DIST	755 ± 393	430 ± 211	<0.1	126 ± 21	115 ± 21	NS	1120 ± 299	730 ± 89	NS	199 ± 21	146 ± 22	0.001
ILE	511 ± 145	361 ± 82	0.1	140 ± 16	129 ± 21	NS	744 ± 202	612 ± 123	NS	255 ± 42	241 ± 64	NS

DUO: duodenum; POX: proximal jejunum; MID: midjejunum; DIST: distal jejunum; ILE: ileum.

**Table 3:** Mass spectrometry analysis of the samples from midjejunum mucosa of IUGR versus NBW piglets.

Protein	Ratio	Fold change	Main biological process
Histone 4	0.26	3.85	Gene uptake
Histone 2B	0.57	1.77	
Putative hexokinase (HKDC1)	0.45	2.23	Carbohydrate metabolism
Hexokinase I (HXKI)	0.66	1.52	
Lamin A/C	0.15	6.8	Chromatin organization
Carbamoyl phosphate 1	0.52	1.92	Protein metabolism
78 kDa glucose-regulated protein precursor	1.27	1.27	Regulator of the unfolded protein response, apoptosis
Mortalin	1.75	1.75	Apoptosis
